# Influence of Age and Genetic Background on Ethanol Intake and Behavioral Response Following Ethanol Consumption and During Abstinence in a Model of Alcohol Abuse

**DOI:** 10.3389/fnbeh.2022.858940

**Published:** 2022-03-28

**Authors:** Silvia Corongiu, Christian Dessì, Elena Espa, Augusta Pisanu, Annalisa Pinna, Daniele Lecca, Sandro Fenu, Cristina Cadoni

**Affiliations:** ^1^Neuropsychopharmacology Section, Department of Biomedical Sciences, University of Cagliari, Cagliari, Italy; ^2^Department of Biomedical Sciences, Institute of Neuroscience, National Research Council of Italy, Cagliari, Italy

**Keywords:** adolescence, alcohol use disorders, genetic vulnerability, Fischer 344 rats, Lewis rats

## Abstract

Genetic background and age at first exposure have been identified as critical variables that contribute to individual vulnerability to drug addiction. Evidence shows that genetic factors may account for 40–70% of the variance in liability to addiction. Alcohol consumption by young people, especially in the form of binge-drinking, is becoming an alarming phenomenon predictive of future problems with drinking. Thus, the literature indicates the need to better understand the influence of age and genetic background on the development of alcohol dependence. To this aim, the inbred rat strains Lewis (LEW, addiction prone) and Fischer 344 (F344, addiction resistant) were used as a model of genetic vulnerability to addiction and compared with the outbred strain Sprague-Dawley (SD) in a two-bottle choice paradigm as a model of alcohol abuse. During a 9-week period, adolescent and adult male rats of the three strains were intermittently exposed to ethanol (20%) and water during three 24-h sessions/week. Adult and adolescent SD and LEW rats escalated their alcohol intake over time reaching at stable levels, while F344 rats did not escalate their intake, regardless of age at drinking onset. Among adolescents, only F344 rats consumed a higher total amount of ethanol than adults, although only SD and LEW rats escalated their intake. Adult LEW rats, albeit having a lower ethanol consumption as compared to SD rats but greater than F344, showed a more compulsive intake, consuming higher amounts of ethanol during the first hour of exposure, reaching a higher degree of ethanol preference when start drinking as adolescents. Behavioral analysis during the first hour of ethanol consumption revealed significant strain differences, among which noticeable the lack of sedative effect in the LEW strain, at variance with F344 and SD strains, and highest indices of withdrawal (most notable jumping) in LEW rats during the first hour of abstinence days. The present results underscore the importance of individual genetic background and early onset of alcohol use in the progression toward abuse and development of alcohol addiction.

## Introduction

The transition from use to abuse of licit and illicit substances can be due to several interacting factors. Among these, genetic background and age at the time of first exposure to the substance have been shown to contribute significantly. Evidence from twin studies indicates that genetic background accounts for 50–60% of the variance in liability to alcohol dependence in humans (Heath and Martin, 1994; True et al., 1999; Heath et al., 2001; Agrawal and Lynskey, 2008; Kendler et al., 2008; Grant et al., 2009; Agrawal et al., 2012). Moreover, early onset of alcohol use, especially in the form of binge-drinking, is concerning given that it has been correlated with alcohol abuse and dependence and other disorders later in life (Grant and Dawson, 1997; DeWit et al., 2000; Dawson et al., 2008; Sartor et al., 2009; Spear, 2011; Liang and Chikritzhs, 2013; Yuen et al., 2020).

Although environmental and genetic factors contribute to the etiology of Alcohol Use Disorder (AUD), a more thorough understanding as to why some individuals become addicted while others do not presumably requires the detailed study of differential actions of ethanol on brain structure/function. The old theory on ethanol pharmacology as an unspecific pharmacological agent has been replaced by detailed pharmacological studies that show several specific molecular targets for ethanol (Abrahao et al., 2017). Among the different effects of alcohol, at multiple molecular targets, the effects on dopamine (DA), endocannabinoid, and opioid systems appear of critical importance for the rewarding and reinforcing properties of alcohol (Spanagel, 2009; Abrahao et al., 2017). Clearly, understanding the etiology of AUD has to keep into account likely different adaptive changes occurring in the above systems following repeated exposure to ethanol. Due to both technical and ethical reasons, this is difficult to study in humans, and therefore, we decided, more than a decade ago, to investigate differences in DA transmission functionality in an animal model of genetic vulnerability to addiction, the inbred rat strains Fischer 344 (F344) and inbred Lewis (LEW). Studies by several groups suggest that the genetic vulnerability of LEW strain to addiction, as compared with the F344 strain, is the result of differences in several neurotransmitter systems in basal conditions but, more importantly, following exposure to drugs of abuse and stress (see Cadoni (2016) for a review). We have previously shown that the greater sensitivity of LEW strain to drugs of abuse, compared with F344 one, might be the result of its greater mesolimbic DA transmission responsiveness to these drugs. Indeed LEW rats, as compared with F344 strain, display higher DA release in the nucleus accumbens shell and core in response to morphine, nicotine, cocaine, and Δ^9^-tetrahydrocannabinol (Cadoni and Di Chiara, 2007; Cadoni et al., 2009, 2015). More notably, LEW strain shows different adaptive changes following repeated exposure to drugs of abuse, retaining (if exposed at adulthood) or even increasing (if exposed at adolescence) their DA response in the nucleus accumbens shell to drug challenge (Cadoni et al., 2015; Cadoni et al., 2020; Lecca et al., 2020). These differences might contribute to the proneness of LEW strain rats to develop higher drug intakes as seen in self-administration paradigms (Picetti et al., 2010; Picetti et al., 2012; Lecca et al., 2020). In addition to being more sensitive to several drugs of abuse, LEW rats appear to be high alcohol preferring, reaching higher rates of intake when compared with F344 rats (Li and Lumeng, 1984; Suzuki et al., 1988; Wilson et al., 1997), and showing neurochemical characteristics (firing modality of dopaminergic neurons, D2 receptors density, etc.) similar to other rat strain lines selected in the world to model alcohol abuse (Minabe et al., 1995; Flores et al., 1998).

Thus, in order to further investigate the influence of genetic background and age of first exposure on the development of alcohol dependence, we compared the intakes of LEW and F344 rats, as an animal model of genetic vulnerability to drug addiction, given intermittent access to high ethanol concentration (20% V/V) in a two-bottle choice test. This protocol has been gaining popularity for animal modeling of alcohol abuse, because, given the repeated cycles of abstinence from alcohol, it leads to escalation of voluntary ethanol intake and preference, thus, mimicking the human condition (Wise, 1973; Simms et al., 2008; Carnicella et al., 2014). To further compare the above genotypes with a group representative of a variable genetic background, we used the outbred rat strain Sprague-Dawley (SD). Analysis of alcohol intake (as daily, weekly, and cumulative intake in 9 weeks) has been expanded to a careful analysis of behavior following ethanol intake and during withdrawal days. Indeed, behavioral analysis following ethanol intake, such as behavioral activation or sedation, and, more importantly, following abstinence days, might provide useful information about the rewarding and motivational value of alcohol helpful for further investigations. Thus, the first aim of the study was to evaluate the influence of the genetic background on ethanol intake and thus, likely, on the development of alcohol abuse and second to clarify the influence of age at first exposure depending on the genetic background. Indeed, even if human studies suggest that age of onset of alcohol use is associated with the risk to develop AUD later in life, recent systematic reviews suggest that this association is less consistent and might be driven by confounding factors, such as the history of alcohol problems in the family, preceding mental health problems, socioeconomic status, or genetic background (Maimaris and McCambridge, 2014; Marshall, 2014; Kuntsche et al., 2016). Even animal studies on this issue are not always consistent (Labots et al., 2018; Spear, 2018; Towner and Varlinskaya, 2020; Mugantseva et al., 2021) and therefore, further investigation is needed to help in clarifying the role of causality between early alcohol onset and increased risk of later AUD.

## Materials and Methods

### Animals

Male outbred SD, LEW, and F344 rats (Charles River, Calco, Italy) of 5 weeks (30–35 postnatal day, PND) or 9 weeks (58–63 PND) of age at arrival were group housed and left to acclimatize to the new housing conditions for 1 week, under standard conditions of temperature (23°C) and humidity (60%) and a 12 h light-dark cycle (light on 08:00 a.m.) with food and water *ad libitum*. Thereafter, they were single housed in polycarbonate cages (480 mm × 265 mm × 210 mm, mod. 2154F, Tecniplast S.p.A., Buguggiate, Varese, Italy) with matching type wire lids. Thus, at the beginning of ethanol exposure, rats were mid-adolescents (6 weeks of age, 35–42 PND) or adults (10 weeks of age, 65–70 PND). This age for adolescent rats has been intentionally selected because our previous study indicates this age as the most sensitive to the DA releasing effects by drugs of abuse (Corongiu et al., 2020). A total of 101 rats were used, 50 adults and 51 adolescents. They were randomly assigned to the ethanol or water control group. All experimental procedures have been carried out in accordance with the European Council directive (2010/63/UE L 276 20/10/2010) and with the guidelines approved by the Ethical Committee of the University of Cagliari (OPBA).

### Intermittent Alcohol Access Procedure

Adult or adolescent rats, individually housed, were exposed to a two-bottle choice regimen (water vs. ethanol 20% V/V) with an intermittent alcohol access for three 24-h sessions per week (Monday-Wednesday–Friday) for a total of 9 weeks. Access to alcohol started at the beginning of the light cycle (08:00) and ended after 24 h. When alcohol was not available, both bottles were filled with water and the amount consumed was recorded for each side. The left and right positions for alcohol and water bottles were switched between sessions to avoid any side bias effect on intake. Both water and alcohol were made available through graduated 190 ml capacity polycarbonate bottles (ACBT0152) equipped with stainless steel caps (ACCP0111) (Tecniplast S.p.A., Buguggiate, Varese, Italy). The bottles were refilled with fresh solutions at every session. On the day of alcohol availability, after having recorded water consumption, animals were weighed and returned to their home cages and soon after the two bottles (water and alcohol) were placed in the cage. Alcohol and water intake in 24 h was monitored by weighing the bottles (accuracy 0.1 g) and then intake referred to each animal as g/kg of body weight. In addition, at sessions 1, 9, 12, and 19, alcohol intake after the first hour of exposure (T1) was recorded as an index of the animal motivation to drink at the beginning and following the abstinence day. Possible fluid spillage was calculated by using multiple bottles filled with water and 20% ethanol, positioned in empty cages. The mean of this passive leakage was subtracted from the weight change of individual fluid bottles of experimental subjects at each analysis point. Control animals were kept in the same conditions but both bottles contained water. This group served as a control for water intake.

### Behavioral Recording During First Hour of Ethanol Exposure or Withdrawal

During the first hour of alcohol exposure and the first hour of withdrawal day of the sessions indicated below, the animal behavior was recorded and then analyzed and scored as the percentage of time spent in each behavioral category in 60 min (after ethanol exposure: sedation, locomotion, licking, sniffing, and rearing; after ethanol withdrawal: jumping, paw treading, locomotion, head burying, gnawing). Behavioral effects were recorded during the first week of exposure (1st and 3rd sessions), third week (9th session), fifth week (13th session), and seventh week (19th session). Withdrawal score was recorded the day after the 8th and 19th sessions but since there were no substantial differences between sessions scores have been pooled.

### Statistics

All data were expressed as mean ± SEM. Statistical analysis was carried out by Statistica for Windows (Version 7.0 Statsoft, Tulsa, OK, United States). Daily and weekly ethanol intake data (g/kg) were analyzed by three-way and two-way ANOVA for repeated measures to unveil significant differences, with age and strain as between factors and sessions or weeks as within measure. One way and two-way ANOVAs were applied to cumulative ethanol and water intake, respectively, with strain, age, and ethanol exposure as independent factors. Differences in ethanol preference were analyzed within each age group by two-way ANOVA for repeated measures with strain as between factor and weeks as within factor. Three-way ANOVA was applied to ethanol intake at T1 with strain and age as between factors and sessions as within factors. Behavioral scores were analyzed by one-way ANOVA for each behavioral item with strain as between factor. Results showing significant effects following ANOVA were subjected to Tukey’s *post hoc* test. The significance level was set at *p* < 0.05.

## Results

### Alcohol Consumption in Adult and Adolescent Rats

**FIGURE 1:**
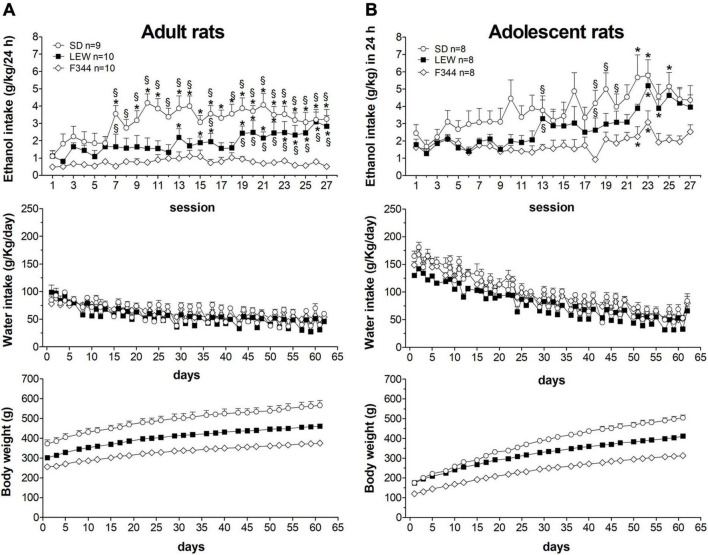
Daily pattern of alcohol intake. Alcohol intake (g/kg/day) by adults **(A)** and adolescents **(B)** onset rats of Sprague-Dawley (SD), Lewis (LEW), and Fischer 344 (F344) strains and daily water intake (g/kg/day) during ethanol regimen. Lower panels show the body weight curves for the three strains during the entire experimental period. Results are expressed as means ± SEM. *p < 0.05 vs. first session within the same strain and §*p* < 0.05 vs. the corresponding value in the F344 strain by two-way ANOVA for repeated measures followed by Tukey’s *post hoc* test.

Figure 1 shows the daily consumption of alcohol and water in the three strains of rats together with daily water intake and body weight gain curve during the alcohol exposure regimen. Three-way ANOVA for repeated measure was applied to ethanol intake that revealed significant main effects of strain, age, and time (F_strain_(2,47) = 19.58, *p* < 0.00001; F_*age*_(1,47) = 9.04, *p* < 0.01; F_time_(26,1222) = 13,16, *p* < 0.000001) and a significant interaction of time × strain (F_52,1222_ = 3.21 *p* < 0.00001) and time × age (F_26,1222_ = 3.32, *p* < 0.00001) but not for strain × age and time × strain × age (*p* > 0.05). *Post hoc* analysis revealed that overall adolescents drink more ethanol than adults (*p* < 0.01), SD drink more than LEW and F344 (*p* < 0.01, *p* < 0.001), and LEW more than F344 (*p* < 0.01). To better highlight differences over time between strains, we performed a two-way ANOVA for repeated measures within each age group which showed significant strain differences in ethanol intake [*adults*: F_strain_(2,26) = 13.71, *p* < 0.0001; F_time_(26,676) = 6.55, *p* < 0.0001; F_strain x time_ (52,676) = 2.73, *p* < 0.0001; *adolescents*: F_strain_(2,21) = 6.86, *p* < 0.01; F_time_(26,546) = 13.55, *p* < 0.0001; F_strain x time_ (52,546) = 1.52, *p* < 0.05]. Tukey’s *post hoc* analysis showed that SD and LEW rats, both adult and adolescent, increase their alcohol intake over time while F344 rats do not, no matter if adults or adolescents. Adolescent rats start drinking greater amounts of alcohol compared with adults (SD: 2.45 ± 0.5 vs. 1.1 ± 0.32 g/kg/day; LEW: 1.79 ± 0.31 vs. 1.1 ± 0.30; F344: 1.64 ± 0.26 vs. 0.48 ± 0.13), although there were no significant differences between strains at least in the first 3 weeks of exposure (from 1st to 12th session, Figure 1B upper panel).

**FIGURE 2:**
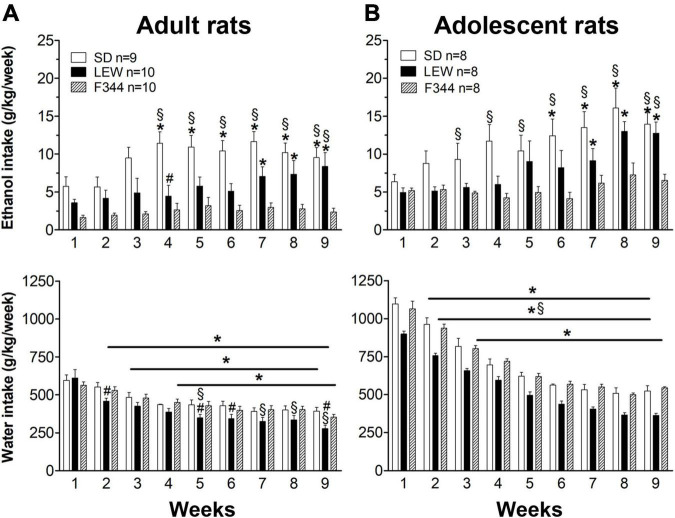
Weekly pattern of ethanol and water intake. Weekly ethanol and water intake (g/kg/week) in the two age groups **(A)** adults and **(B)** adolescents of the three strains. Results are expressed as means ± SEM. *p < 0.05 vs. first week within each strain, #*p* < 0.05 vs. Sprague-Dawley (SD) strain, §*p* < 0.05 vs. Fischer 344 (F344) strain by two-way ANOVA for repeated measures followed by Tukey’s *post hoc* test.

The same analysis revealed that SD rats consume greater alcohol amounts than LEW and F344 and LEW greater than F344 strain when adult (Figure 1A upper panel), but these differences fail to emerge when they were adolescent at the onset of ethanol exposure, at least in the first 3 weeks of exposure (Figure 1B upper panel). Analyzing alcohol consumption as weekly intake by two-way ANOVA for repeated measures (Figure 2) highlights better the escalation in ethanol intake in SD and LEW strains, but not in F344 one, in both age groups. Water intake decreased over time in the three strains in both age groups [*adults*: F_time_(8,208) = 70.10, *p* < 0.0001; *adolescents*: F_time_(8,168) = 335,23, *p* < 0.00001] but to a greater extent in LEW rats [*adults*: F_strain_(2,26) = 2.3, *p* = 0.11; F_strain x time_(16,208) = 2.59, *p* < 0.01, *post hoc p* < 0.05, see Figure 2A for details; *adolescents*: F_strain_(2,21) = 15.54, *p* < 0.0001; F_strain x time_(16,168) = 1.021, *p* = 0.4], and more in adolescent than adult rats [F_*age x time*_(8,128) = 16.13, *p* < 0.0001] given that adolescent rats drink more water than adults (Figures 2A,B).

**FIGURE 3:**
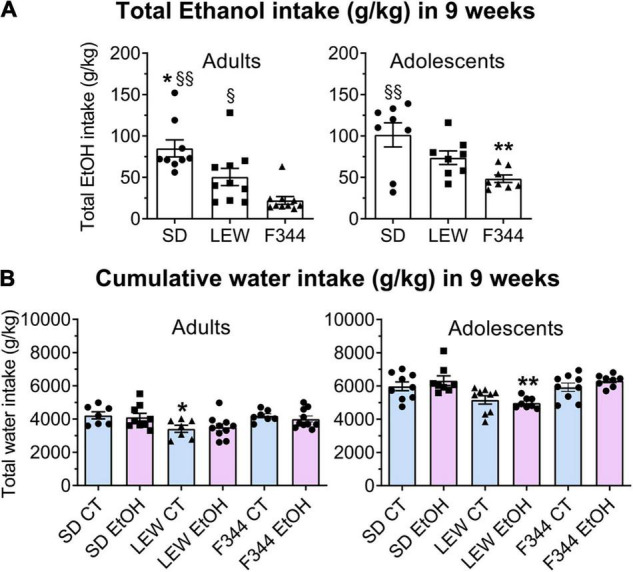
Cumulative ethanol and water intake in 9 weeks. **(A)** Total ethanol intake (g/kg/9 weeks) in adult and adolescent groups of the three strains. Data are expressed as means ± SEM. *p < 0.05 Sprague-Dawley (SD) vs. Lewis (LEW), §§*p* < 0.001 SD vs. Fischer 344 (F344), §*p* < 0.05 LEW vs. F344, **p < 0.01 F344 adolescents vs. adults by one-way ANOVA followed by Tukey’s *post hoc* test. **(B)** Cumulative water intake (g/kg/9 weeks) in adult and adolescent groups of the three strains. Water intake by control rats (CT) is reported for comparison with ethanol-exposed rats (EtOH). *p < 0.05 LEW CT vs. SD and F344 CT; **p < 0.001 LEW EtOH vs. SD and F344 EtOH by two-way ANOVA followed by Tukey’s *post hoc* test.

One-way ANOVA within each strain applied to cumulative alcohol intake in the 9-week period did not reveal any significant difference between adults and adolescents in SD and LEW rats (F_1,15_ = 0.94, *p* = 0.34; F_1,16_ = 2,72, *p* = 0.11, respectively), although generally daily ethanol intake in adolescent individuals was higher than adult ones (mean intake g/kg/day during last week SD: 4.64 ± 0.25 vs. 3.18 ± 0.06; LEW: 4.26 ± 0.20 vs. 2.79 ± 0.19; F344: 2.19 ± 0.18 vs. 0.62 ± 0.08), while adolescent F344 rats show the greater total amount of alcohol consumed compared with their adult counterparts, as clearly apparent in Figure 3A (F_1,16_ = 15.68, *p* < 0.01). Analysis of cumulative ethanol intake between strains in adults shows that SD ethanol intake was greater than LEW and F344 (F_2,25_ = 12.09, *p* < 0.001; *post hoc p* < 0.05 and *p* < 0.001) and LEW intake greater than F344 (*p* < 0.05), while in adolescent groups, only SD ethanol intake was greater than that one of F344 rats (F_2,21_ = 6.99, *p* < 0.01; *post hoc p* < 0.01).

Comparison of cumulative water intake between controls and ethanol exposed group of each strain and age group (Figure 3B) revealed no difference between ethanol and control groups in the total amount of water intake (adults: SD F_1,14_ = 0.11, *p* = 0.74; LEW: F_1,15_ = 0.08, *p* = 0.77; F344: F_1,15_ = 0.57, *p* = 0.46; adolescents: SD F_1,15_ = 0.7, *p* = 0.4; LEW: F_1,15_ = 0.3, *p* = 0.5; F344: F_1,15_ = 1.7, *p* = 0.2), indicating that even if ethanol exposed groups reduce their water intake during alcohol sessions they drink more water during days in which alcohol was not available.

**FIGURE 4:**
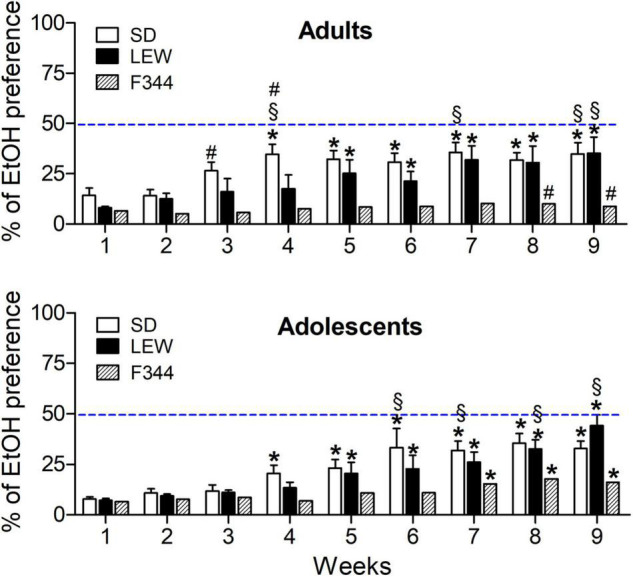
Alcohol preference expressed as percentage of total fluid intake in adult and adolescent groups of the three strains. Data are expressed as means ± SEM. *p < 0.05 vs. preference in the first week within each strain; §*p* < 0.05 vs. corresponding value in the Fischer 344 (F344) strain; # *p* < 0.05 vs. the corresponding value in the adolescent group of the same strain by two-way ANOVA for repeated measures followed by Tukey’s *post hoc* test.

Two-way ANOVA applied to cumulative water intake revealed that among adult controls, LEW rats drink less than SD and F344 rats (F_2,18_ = 5.79, *p* < 0.01), while among adolescents, ethanol exposed LEW rats drink significantly less water than the other two strains (F_2,21_ = 15.54, *p* < 0.0001, *post hoc p* < 0.001), with no difference between control groups (F_2,24_ = 3.2, *p* = 0.05), at variance with what observed in adult rats. These results are consistent with the higher ethanol preference observed in the LEW strain (Figure 4, lower panel).

The analysis of alcohol preference, calculated as a percentage of ethanol consumption on the total amount of liquid drunk by each rat, revealed that ethanol preference in LEW and SD strain increases over time [F_strain x time_ (16,208) = 3.04, *p* < 0.001, see Figure 4 legend for *post hoc* details] reaching the same amount of ethanol preference and being higher than that one of F344 rats in the adult group (Figure 4, upper panel). A similar increase of ethanol preference was observed in adolescents, but a significant increase of preference was observed also in F344 strain, while, at the end of alcohol exposure, only LEW strain showed an ethanol preference higher than F344 strain [F_strain x time_(16,168) = 2.95, *p* < 0.001, see Figure 4 legend for *post hoc* details), reaching almost 50% of ethanol preference.

**FIGURE 5:**
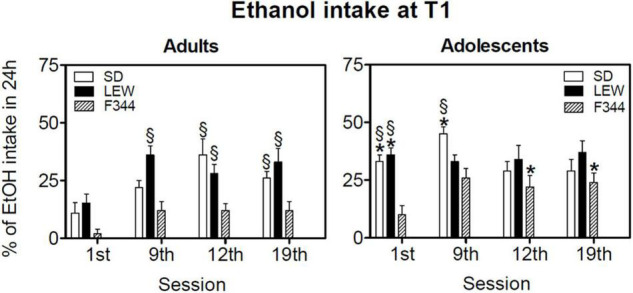
Ethanol intake during the first hour of the drinking session (T1). Data are expressed as means ± SEM. *p < 0.05 vs. the corresponding value in adult rats, §*p* < 0.05 vs. corresponding value of Fischer 344 (F344) rats by three-way ANOVA for repeated measures followed by Tukey’s *post hoc* test.

Figure 5 shows the percentage of alcohol intake during the first hour of exposure at different time points of ethanol exposure (1st, 9th, 12th, and 19th sessions). Three-way ANOVA for repeated measures revealed that adult LEW rats consume in general a greater percentage of their daily intake than F344 strain during the first hour of exposure, while this difference fades in adolescent groups [F_strain x age x session_ (6,141) = 3.49, *p* < 0.01, see Figure 5 legend for *post hoc* comparisons between strains and ages].

### Behavioral Effects After Ethanol Exposure and During Withdrawal

**FIGURE 6:**
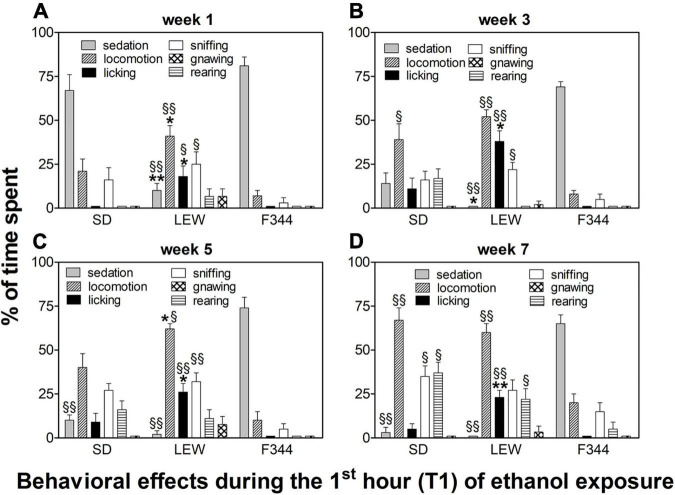
Behavioral effects in adult onset rats during the first hour of the drinking session. Data are expressed as means ± SEM. **(A)** first week: data are referred to the first and third sessions of exposure. **(B)** third week: data are from the 9th session. **(C)** fifth week: data are from the 13th session; and **(D)** seventh week: data are from the 19th session. *p < 0.05 and **p < 0.001 Lewis (LEW) vs. Sprague-Dawley (SD), §*p* < 0.05 and §§*p* < 0.001 vs. corresponding value in Fischer 344 (F344) strain by one-way ANOVA followed by Tukey’s *post hoc* test.

**FIGURE 7:**
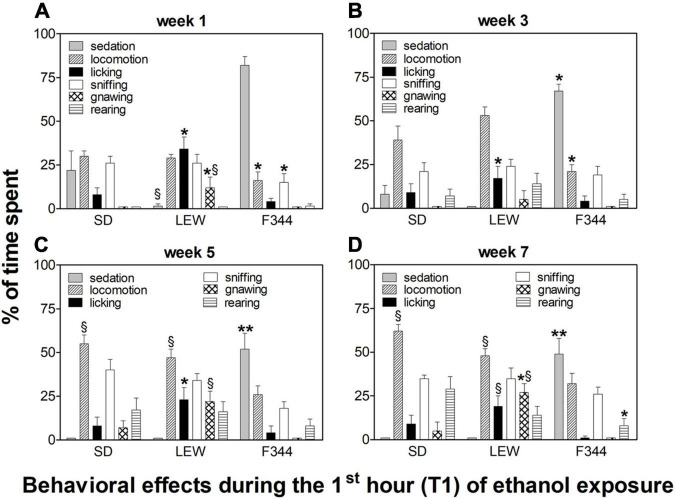
Behavioral effects in adolescent onset rats during the first hour of the drinking session. Data are expressed as means ± SEM. **(A)** first week: data are referred to the first and third session of exposure; **(B)** third week: data are from the 9th session; **(C)** fifth week: data are from the 13th session; and **(D)** seventh week: data are from the 19th session. *p < 0.05 Lewis (LEW) vs. Sprague-Dawley (SD) and Fischer 344 (F344) strain, **p < 0.001 F344 vs. LEW and SD, §*p* < 0.05 SD and LEW vs. F344 strain by one-way ANOVA followed by Tukey’s *post hoc* test.

Behavioral observations made during the first hour of ethanol exposure revealed different patterns of effects depending on the strain and onset age (Figures 6, 7).

In adult rats, following the first 3 sessions of alcohol exposure, there was a significant difference between strains. While SD and F344 rats manifested a profound sedation following ethanol intake (F_2,26_ = 36,72, *p* < 0.0001; *post hoc p* < 0.001), LEW rats showed a behavioral activation characterized by locomotion, sniffing, and hedonic reactions, such as genitals licking (and associated penile erection) and licking of the cage walls (*locomotion*: F_2,26_ = 10.09, *p* < 0.001; *licking*: F_2,26_ = 8.11, *p* < 0.01; *sniffing*: F_2,26_ = 3.41, *p* < 0.05), this last item being completely absent in the other two strains (Figure 6A, see figure legend for *post hoc* significance). Following multiple ethanol exposures, a locomotor sensitization appeared in SD strain (F_3,51_ = 3.08, *p* < 0.05, *post hoc p* < 0.05 seventh week vs. first, third, and fifth weeks) accompanied by a total disappearance of sedation, which was still present in F344 strain even during the seventh week (F_2,26_ = 149, *p* < 0.0001, see Figure 6 legend for *post hoc* comparisons). Repeated exposure to ethanol induced the appearance of hedonic reactions in SD rats (licking), still lacking in F344 strain (Figures 6B–D). In LEW rats, there was not an increase in locomotor activity but the appearance of confined sniffing and licking still higher when compared with the other two strains (third week: F_2,26_ = 15.53, *p* < 0.0001; fifth week: F_2,26_ = 10.78, *p* < 0.001; seventh week: F_2,26_ = 15.78, *p* < 0.0001, see Figure 6 legend for *post hoc* comparisons).

Behavioral effects in adolescent rats were characterized by an almost absent sedation in SD rats (F_1,15_ = 8.87, *p* < 0.01, *post hoc p* < 0.05), but still present in the F344 strain, although there was a trend toward reduction when compared with the adult counterpart (F_3,48_ = 2.7, *p* = 0.05). Adolescent LEW rats displayed a reduction in locomotion when compared with the adult counterpart (F_1,16_ = 6.15, *p* < 0.05; *post hoc p* < 0.05) but showed the appearance of stereotypies (confined sniffing and gnawing), Figure 7.

**FIGURE 8:**
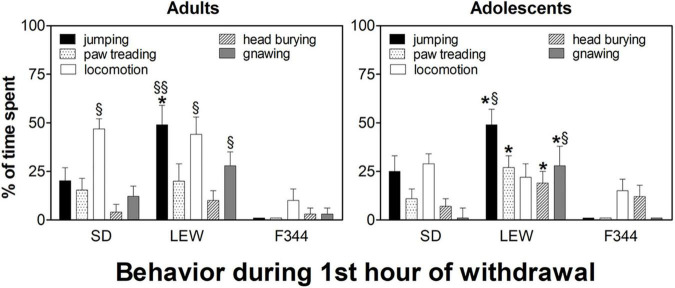
Behavioral reactions during the first hour of withdrawal in the three strains in adult and adolescent onset rats. Data are expressed as means ± SEM. Results from the day after the 8th and 19th drinking sessions were pooled since no significant difference was observed. *p < 0.05 Lewis (LEW) vs. Sprague-Dawley (SD), §*p* < 0.05 LEW and SD vs. Fischer 344 (F344), §§*p* < 0.001 LEW vs. F344 by one-way ANOVA followed by Tukey’s *post hoc* test.

Analysis of behavior during the first hour of withdrawal showed a profound difference between strains (Figure 8). In the adult group, LEW strain manifested significant “craving like” reactions (mainly jumping, locomotion, and gnawing) when compared with the other two strains, notably vs. F344 strain. Time spent in jumping by the LEW strain was higher than that one by the other two strains (F_2,26_ = 10.02, *p* < 0.001) as well as locomotion, and gnawing time spent was higher than that one by the F344 strain (*locomotion*: F_2,26_ = 7.98, *p* < 0.01; *gnawing*: F_2,26_ = 3.75, *p* < 0.05). In the adolescent group, differences between strains were similar to those observed in the adult group, with the only difference of a reduced locomotion in SD rats (F_1,15_ = 5.14, *p* < 0.05; *post hoc p* < 0.05) when compared with the adult counterpart (Figure 8).

It is worth noting, in regard to withdrawal reactions, that animals manifesting greater “craving like” reactions drank greater amounts of water from the bottle placed on the same side where, the previous day, the alcohol bottle was placed (data not shown).

## Discussion

The results presented highlight differences in alcohol intake and behavioral effects induced by repeated and intermittent alcohol consumption, and alcohol withdrawal, due to age of first exposure and genetic background of the individual. The intermittent alcohol access paradigm utilized in this study shows how genetic background may affect drinking pattern. While SD and LEW strains display an escalation of alcohol intake, F344 strain does not, thus, confirming previous reports for the SD strain (Li et al., 2010; Bito-Onon et al., 2011; Li et al., 2011), and adding new evidence for the inbred rat strains LEW and F344. Comparison of the age groups revealed that while in the adult onset group, differences between strains emerge after the first 2 weeks of exposure, in the adolescent onset group, differences due to genetic background emerge later on, when the rats become adults. This pattern of results is consistent with what is observed in humans. Individual differences in alcohol (but also in other substances of abuse) initiation and patterns of use are reported to be strongly influenced by social and familial environmental factors, while later levels of use appear to be strongly influenced by genetic factors (Kendler et al., 2008; van Beek et al., 2012). Although adolescent rats start drinking more than adults, there were no statistical differences between adult and adolescent SD and LEW rats in the total amount of alcohol intake during the entire period (9 weeks) of exposure. However, F344 strain, considered an addiction resistant strain, on the basis of several previous findings (Cadoni, 2016), although not escalating alcohol intake over time (at least in a period of 9 weeks as in the present study) displays a significantly higher total ethanol intake if starting to drink as adolescent, shows an increase in ethanol preference in the last 2 weeks of exposure, and an increased ethanol intake at T1. This would suggest that even individuals resistant to develop AUD might be at risk if they start to drink as an adolescents.

One of the reasons why the adolescent onset of drinking seems to affect more the F344 strain (addiction resistant) than LEW (addiction prone) and SD ones might be the different impact of ethanol on developing brain of these strains. It is well known that during adolescence, there is a rearrangement of several neuronal circuitries, with changes in number and subunits composition of different neurotransmitter receptors in different brain areas (Spear, 2000; Brenhouse and Andersen, 2011). Indeed, there are several fundamental differences between strains, in particular, between F344 and LEW rats (Cadoni, 2016), in neurotransmitter systems targeted by alcohol (gabaergic, dopaminergic, serotonergic, cannabinoidergic) which might play a role in the impact of ethanol on adolescent brain of each strain. For examples, adolescent THC (Δ^9^-tetrahydrocannabinol) exposure affects differently DA transmission in SD, LEW, and F344 rats, respectively, by decreasing, leaving unaffected, and increasing DA transmission responsiveness in the nucleus accumbens shell of these strains (Cadoni et al., 2008; Cadoni et al., 2015). Similar results were obtained after adolescent nicotine exposure, and different adaptive changes in DA transmission have been observed depending on adolescent or adult drug exposure (Cadoni et al., 2020). It is conceivable that if similar adaptive changes occur following repeated ethanol exposure too, they could affect differently drinking pattern in each strain. Another point to be considered in this regard is the fact that SD strain is an outbred strain, at variance with F344 and LEW, and therefore, the greater variability in the SD strain might have masked a likely effect of early onset in some individuals but not in others, while this variability is almost absent in the inbred strains. Even if there were not differences between adolescent and adult rats of the SD and LEW strains, analysis of ethanol preference in the two age groups of the three strains revealed that LEW adolescent rats display the highest ethanol preference the last week of exposure, reaching on average almost 50% of ethanol preference, with some animals drinking nearly exclusively alcohol.

The fact that SD rats, both adults and adolescents, drink more than LEW rats (considered an addiction-prone strain) might be explained by a greater sensitivity of the LEW strain to ethanol rewarding effect and/or different ethanol pharmacokinetics in the two strains. Indeed, previous findings in SD, LEW, and F344 rats have shown LEW rats having a slower alcohol metabolism, thus, leading to higher blood ethanol concentrations (BEC) at differing time points following alcohol exposure when compared with SD (Bito-Onon et al., 2011), but also with F344 rats (Suzuki et al., 1988; Roma et al., 2007; Roma et al., 2008). Moreover, on the basis of our previous studies (Cadoni and Di Chiara, 2007; Cadoni et al., 2015; Cadoni et al., 2020) showing a higher sensitivity of the LEW strain to the DA releasing properties of different drugs of abuse and different adaptive changes in mesolimbic DA transmission following repeated drug exposure, it can be hypothesized that similar pattern of response might occur following ethanol too. Both of these reasons might explain why LEW rats drink little less than SD ones.

In the present study, we compared also cumulative water intake between controls and ethanol group of each strain and age group. This analysis revealed a difference between adult and adolescent groups with adolescent rats drinking a larger amount of water as compared to adults. This is a general feature in human infants/adolescents, and presumably in rodents too, and is due to high requirements for water of infants/adolescents to maintain an adequate body composition (Iglesia et al., 2015). This high requirement can be in part explained by a proportionally higher body water content than adults. Although we did not observe differences within each age between the water and ethanol group of the three strains, we detected a reduced water intake in control adult rats of the LEW strain and a reduced water intake in adolescent ethanol-exposed rats of the same strain. This would indicate that the reduced water intake in adult control LEW rats cannot be explained only by body mass, given that F344 rats although having a lower body weight consume the same amount of water as SD strain, but other factors might be involved. On the other hand, we observed a reduced water intake in adolescent LEW ethanol-exposed rats, which would be consistent with an increased ethanol preference in this strain of rats.

Ethanol intake during the first hour of alcohol availability after the day of abstinence (expressed as a percentage of 24 h intake) is somehow a measure of individual compulsiveness to consume alcohol, and it is related to the state of withdrawal experienced by the animal during the sessions following the first one. In general, adolescent rats consume more alcohol than adults during the first session, and this is likely correlated to a different motivation (high novelty seeking behavior in adolescents), but also to alcohol effects experienced, given that the first exposure usually produces sedative effects in adults while producing activating effects in adolescents, as it has been reported in the result section (see also Figures 6, 7). Although, as described before, SD showed the highest rates of alcohol consumption, they are not always compulsive as the LEW strain, consuming most of their daily intake during the dark phase.

Analysis of behavior during the first hour following ethanol consumption revealed significant differences between strains and age groups. In the adult group, while SD and F344 strains showed a prevalent sedative effect at the first week, LEW rats displayed a significant behavioral activation characterized by locomotion, sniffing, and licking. A low sedative response following alcohol intake is considered a risk factor to develop AUD later in life in humans and in laboratory animals (Crews et al., 2016). Moreover, behavioral effects observed following ethanol intake would suggest a greater DA transmission stimulation (Wise and Bozarth, 1987) and a greater hedonic effect (as shown by the presence of licking). Following repeated exposures to ethanol, SD rats developed behavioral sensitization, manifested as tolerance to the sedative effect of alcohol, and increased time spent in locomotor activity as previously observed in this strain (Wilson et al., 1997). These striking differences between strains in sedative effects might be due to differences between strains in gamma-aminobutyric acid (GABA) levels, GABA-A receptors distribution and/or functionality (Cadoni, 2016), and different adaptive changes in DA transmission in striatal areas (ventral and dorsal striatum). Indeed, previous studies have shown that LEW and F344 strains differ not only in basal levels of GABA and Glutamate in NAc but also in response to acute ethanol (Selim and Bradberry, 1996) and cocaine reinstatement in a self-administration paradigm (Miguéns et al., 2011).

The behavior observed in the adolescent group following alcohol consumption revealed a marked reduction of sedative effect in SD strain (almost absent) and a modest reduction in the F344 strain. The lower sedative effect observed in adolescent rats following ethanol consumption is a general hallmark of adolescence, both in humans and rodents (Little et al., 1996; Moy et al., 1998; Novier et al., 2015; Spear, 2018), and it appears correlated to developmental changes of GABA-A receptors, and other receptor types too (N-methyl-D-aspartate [NMDA], DA) (Crews et al., 2007; Novier et al., 2015). Therefore, sedative or activation effects do not appear to be correlated to the amount of ethanol intake in this study, as previously observed in other studies showing that adolescent rats recover the righting response following a sedative dose of ethanol in spite of higher brain alcohol levels than adults (Silveri and Spear, 1998). F344 rats displayed also a behavioral activation (locomotion, sniffing, and rearing), almost absent in adult counterparts, indicating a different impact on neurotransmitter systems mediating alcohol effects in the adolescent brain (Spear, 2018). Adolescent LEW rats, on the contrary, do not display any sedative effects as previously observed in adult counterpart but, at variance with SD strain, show a reduced locomotor activity compared with adults, and appearance of more stereotypies, such as confined sniffing and gnawing, this last one being significantly higher than SD and F344 rats. The appearance of stereotyped activity is usually correlated to an intense DA receptors activation, especially in striatal areas (Kelly et al., 1975; Bordi and Meller, 1989; Delfs and Kelley, 1990). This difference might be due to a different impact of alcohol on DA transmission between adults and adolescents and/or to different adaptive changes in DA transmission following alcohol exposure during the critical period of adolescence. Indeed, our previous studies on adolescent rats have shown a higher sensitivity, in terms of DA released, to several drugs included opiates (Corongiu et al., 2020) and different adaptive changes in mesolimbic DA transmission following adolescent exposure to cannabinoids, opiates, and nicotine in LEW, F344, and SD rats, as discussed above.

It is worthy of note in this regard that LEW rats are, among several rat lines, the most sensitive to the rewarding and activating effects of nicotine (Cadoni et al., 2009; Chen et al., 2012) and that rats selectively bred for high alcohol consumption/responsivity are also more likely to self-administer nicotine (Deehan et al., 2018), in agreement with human studies, suggesting a common genetic basis for alcohol and nicotine addiction (Cross et al., 2017).

The analysis of behavior during ethanol withdrawal indicates the LEW strain as the most affected and this might be a factor influencing the establishment of alcohol dependence. Indeed, withdrawal syndrome has been implicated as one of the mechanisms for the progression from impulsive to compulsive substance use. In this regard, jumping behavior in rodents, highly expressed by LEW rats in this study during withdrawal, is widely considered as an index of opiates withdrawal intensity and is commonly used to test opiate dependence (Ritzmann, 1981; Kest et al., 2001; Dunn et al., 2019). Reasons for this heightened vulnerability of LEW strain might be searched in the mechanism of action of alcohol and LEW characteristics in DA and opioid systems (Cadoni, 2016). In fact, the reinforcing and rewarding effects of alcohol are primarily mediated by the DA mesolimbic and opioid systems (Spanagel, 2009). In addition to stimulate mesolimbic DA transmission, ethanol releases endorphins into the nucleus accumbens (Olive et al., 2001). Therefore, it is likely that repeated alcohol consumption might have induced adaptive changes in opioid receptors similar to those observed after repeated morphine or heroin. It is worthy of note that LEW rats show, in comparison with F344 strain, opposite changes in endogenous opioids following chronic morphine (Cadoni, 2016) and an increased binding to mu-opioid receptors (Sánchez-Cardoso et al., 2007) consistent with a higher score of “craving like” reactions observed in this strain following repeated exposure to heroin (Cadoni et al., 2015), and a similar correlation has been reported in human alcoholics showing higher rates of craving during abstinence (Heinz et al., 2005; Hansson et al., 2019).

A limitation of this study might be due to the experimental condition utilized. The single housed condition of the animals, especially during adolescence, might have affected the results obtained. This choice has been, however, imposed by the need to evaluate individual alcohol intake, but also by the need to perform other biochemical recordings in another group of animals on the same ethanol regimen (microdialysis experiments actually still running). It is well known that isolation is a stressful condition for adolescent individuals (Burke et al., 2017; Walker et al., 2019) which can lead to increased ethanol intake in two-bottle choice paradigms (McCool and Chappell, 2009; Karkhanis et al., 2015), but not in operant self-administration paradigms (Noori et al., 2014). Moreover, stress effects on alcohol consumption might be affected by genetic background (Spanagel et al., 2014) and therefore social isolation might have affected differently the three strains. However, given that we preserved social interaction at least in early adolescence, by group housing animals, and considered that the most critical period for social isolation is between PND 21 and 41, during which rats show most social play behavior (Lesscher et al., 2015; Burke et al., 2017), it could be hypothesized a limited effect on our animals, since most of them were isolated at PND 38–40.

Moreover, it should be emphasized the lack of testing in female rats in the present study. Indeed, it is well recognized that sex/gender may affect substance abuse in general (Becker and Chartoff, 2019; Cornish and Prasad, 2021) and alcohol-induced outcomes, such as drinking pattern, sensitivity to ethanol, anxiety, and neuroinflammation (Robinson et al., 2021). Therefore, further investigation will need to extend the results of the present study to female rats.

Although conclusive remarks will need additional research, by using operant self-administration procedures, to better evaluate the reinforcing and motivational value of alcohol in these strains, nonetheless, the present results highlight the influence of age and genetic background on vulnerability to AUD. On the basis of alcohol consumption (escalation of alcohol intake), it appears that both SD and LEW rats are the most vulnerable to develop alcohol dependence. However, on the basis of behavioral reactions, in particular, during withdrawal days, and alcohol preference, the LEW strain seems the more prone to develop alcohol dependence. On the other hand, although the F344 strain seems to be the less vulnerable strain to develop alcohol dependence, since it does not escalate ethanol intake and does not manifest “craving like” reactions, comparisons between adult and adolescent groups indicate that adolescent F344 rats, increasing their ethanol preference over time, might be at risk to develop alcohol dependence.

In conclusions, the results presented show that the influence of genetic background on alcohol consumption emerges more at adulthood than during adolescence and that adolescent onset of drinking might increase the risk to develop AUD even in genetically less vulnerable individuals.

## Data Availability Statement

The raw data supporting the conclusions of this article will be made available by the authors, without undue reservation.

## Ethics Statement

The animal study was reviewed and approved by Ethical Committee at the University of Cagliari.

## Author Contributions

CC and SF were responsible for the study concept and design. SC, CD, AuP, EE, AnP, and CC contributed to the acquisition of animal data. CC, DL analyzed the data. CC wrote the manuscript. All authors critically reviewed content and approved the final version of the manuscript.

## Conflict of Interest

The authors declare that the research was conducted in the absence of any commercial or financial relationships that could be construed as a potential conflict of interest.

## Publisher’s Note

All claims expressed in this article are solely those of the authors and do not necessarily represent those of their affiliated organizations, or those of the publisher, the editors and the reviewers. Any product that may be evaluated in this article, or claim that may be made by its manufacturer, is not guaranteed or endorsed by the publisher.
